# Notes on the Etiology of Dysentery

**Published:** 1918-05

**Authors:** 


***Notes on the Etiology of Dysentery.*** By C. J. MARTIN and E. F. WILLIAMS (*Brit. Med. Journ.*, 1917, p. 479) and by C. J. MARTIN, KELLAWAY, and E. F. WILLIAMS (*Brit. Med. Journ.*, 1917, p. 480. Abstracted.

Examinations of 217 stools were made. Mannite-fermenting dysentery bacilli were isolated 76 times and non-mannite dysentery bacilli, 47 times. Entamoeba histolytica or its cysts were present in 63 cases, while in 36 no positive organism was detected. The authors report variations in the action of the mannite fermenters on certain carbohydrates. Some of the strains were examined six months after isolation. Out of the 3 which originally fermented saccharose, 2 lost this property. Of the 21 which originally fermented maltose, 5 lost this power, and the other five had acquired it. Other variations are also reported. The production of indol was very variable. 9 lost the power to produce it and 5 acquired it. Nearly one-sixth of the mannite-fermenters were not agglutinated by a Y serum immediately after isolation, but six months later 4 out of 8 of these strains were agglutinated by the same serum.

